# The combined effect of fire and nitrogen addition on biodiversity and herbaceous aboveground productivity in a coastal shrubland

**DOI:** 10.3389/fpls.2023.1240591

**Published:** 2023-08-29

**Authors:** Luyu Qi, Yixin Song, Puyi Zhang, Wenlong Sun, Wei Wang, Shijie Yi, Jing Li, Haifang Liu, Zhenggang Bi, Ning Du, Weihua Guo

**Affiliations:** ^1^ Key Laboratory of Ecological Prewarning, Protection and Restoration of Bohai Sea, Ministry of Natural Resources, School of Life Sciences, Shandong University, Qingdao, China; ^2^ Observation and Research Station of Bohai Strait Eco-Corridor, First Institute of Oceanography, Ministry of Natural Resources, Qingdao, China; ^3^ Shandong Yellow River Delta National Nature Reserve Management Committee, Dongying, China

**Keywords:** aboveground productivity, burned, fungus, nitrogen addition, Yellow River delta

## Abstract

**Introduction:**

Fire and nitrogen (N) deposition each impact biodiversity and ecosystem productivity. However, the effect of N deposition on ecosystem recovery after fire is still far from understood, especially in coastal wetlands.

**Methods:**

We selected a typical coastal shrubland to simulate three N deposition levels (0, 10, and 20 g N m^−2^ year^−1^) under two different burned conditions (unburned and burned) in the Yellow River Delta of North China. Soil properties, soil microbial biodiversity, shrub growth parameters, herbaceous biodiversity, and aboveground productivity were determined after experimental treatments for 1 year.

**Results:**

We found that fire had a stronger influence on the ecosystem than N addition. One year after the fire, shrub growth had significantly decreased, while soil pH, soil electrical conductivity, herbaceous biodiversity, soil microbial biodiversity, and herbaceous aboveground productivity significantly increased. Conversely, a single year of N addition only slightly increased herbaceous aboveground productivity. The combined effect of fire and N addition was only significant for fungus biodiversity and otherwise had minimal influence. Interestingly, we found that herbaceous aboveground productivity was positively associated with fungal community diversity under unburned conditions but not in burned shrublands. Fire showed a great impact on soil parameters and biodiversity in the coastal wetland ecosystem even after a full year of recovery.

**Discussion:**

Fire may also diminish the influence of several belowground factors on herbaceous aboveground productivity, which ultimately reduces recovery and stability. Appropriate N addition may be an effective way to improve the ecosystem productivity in a wetland dominated by shrub species.

## 1 Introduction

Fire is a key ecosystem modifier, affecting soil physicochemical properties as well as biological activities (McLauchlan et al., 2020). Generally, fire is considered destructive to most terrestrial ecosystems (Rousk et al., 2010; Holden et al., 2015; Fernandez-Garcia et al., 2019a; Fernandez-Garcia et al., 2019b). Nitrogen (N) is lost from the ecosystem during and after fire, and loss of more than 50% of N stocks have been reported after fire in a *Eucalyptus pauciflora* forest (Raison et al., 1986). This N loss has consequences for the plant community. Total vegetation biomass and the current year’s net primary productivity are reduced for several years following wildfires because of N limitation (Vogl, 1983). Fire also kills microorganisms living in the topsoil directly because of the high temperature and indirectly influences soil pH, soil moisture, and N content (Akburak et al., 2018). Fire is also a vital ecological factor that maintains biodiversity and ecosystem function and can provide opportunities for the regeneration of understory plants (Aragao and Shimabukuro, 2010; Bento-Goncalves et al., 2012). Fire has been found to promote ecosystem biodiversity in the short term (Webster and Halpern, 2010). For example, burning significantly stimulated herb density (+602.4%), richness (+1.2 species m^−2^), and coverage (+25.5%) over the 3 years after fire in a coniferous-broadleaved mixed forest (Hu et al., 2018b) by reducing canopy thickness and providing gaps for understory species. Despite this, fire can still be incredibly damaging to ecosystems around the world. Burning regimes intended to clear room for cattle destroy forests throughout the global south every day. The influence of fire on vegetation recovery is thus poorly understood, as fire severity, frequency, and initial ecosystem resilience can be highly variable (Abella and Fornwalt, 2015; Pausas and Ribeiro, 2017).

Nitrogen deposition may alleviate ecosystem N limitation following fire, but it has several challenges of its own. By changing the ratio of nutrients in the soil, N deposition can indirectly influence a range of soil properties, such as pH and total nitrogen content (Zhang et al., 2018; Yin et al., 2022), microbial activity and community structure (Vadeboncoeur, 2010; Ramirez et al., 2018), plant growth and community composition (Bobbink et al., 2010; Yin et al., 2022), and ecosystem functions (Perring et al., 2016; He et al., 2021). In most terrestrial ecosystems, N deposition acidifies soils and reduces microbial biodiversity (Aronson and Helliker, 2010; Chen et al., 2018). Aboveground, N deposition reduces plant community diversity by facilitating fast-growing nitrophilous species (Gilliam, 2006; Clark and Tilman, 2008). The study has shown that exogenous nitrogen input can reduce aboveground net primary productivity and biodiversity in a Minnesota tallgrass grassland (Isbell et al., 2013). It is challenging to predict how an ecosystem may respond to nitrogen addition.

While many studies have measured how plant and soil communities respond to either fire or N deposition, few have explored their interaction. N addition and fire may be ultimately beneficial to plant growth, suggesting their interaction should improve community growth and recovery after fire. In fact, some studies report that fire increases soil available N content and enhances plant N uptake and leaf N concentration (Solomun et al., 2021), suggesting that fire promotes plant growth by improving the availability of nitrogen, a mechanism that may be mimicked in N deposition. However, despite similar influences on productivity, N addition and fire generally have opposite influences on biodiversity. For example, in one experiment contrasting their influences, N addition significantly reduced plant diversity within 1 year, while burning did not influence plant diversity at all (Britton and Fisher, 2007). When considering the conservation of these shrubland ecosystems, the contrasting influences of fire and nitrogen on productivity and biodiversity must be considered.

There is considerable debate on whether improvements in biodiversity will always improve productivity. Plant community structure and functional traits are crucial for productivity. If we believe in the niche complementarity effect, which holds that each species has specific niches, increased diversity leads to more thorough coverage of ecological niches, suggesting a diverse complement of plants will ultimately achieve higher productivity (Loreau and Hector, 2001). However, environmental filtering is intense in a number of low-resource habitats, and only a finite number of species can use limited resources efficiently (Ma et al., 2020). This principle is consistent with existing work in resource-poor coastal ecosystems, and studies have shown that N addition enhances tidal marsh plant growth (114% stimulation), particularly at sea levels where plants are most stressed (Langley et al., 2013). The result suggests that most traditional vegetation analysis should consider measures that account for an increase in productivity without a commensurate increase in biodiversity, particularly when researching resource-limited ecosystems. One such approach is the mass ratio hypothesis, which emphasizes the dominant species of a community on the basis that it is responsible for the most ecosystem flux (Lohbeck et al., 2015). A similar approach has been termed the “vegetation quantity hypothesis” (also known as the green soup hypothesis) and holds vegetation “quantity” is more important than “quality”. These quantity-based approaches have seen success in recent years, and research has demonstrated that ecosystem productivity can be affected by vegetation quantity (i.e., density, represented by stand biomass or basal area per unit area) (Lohbeck et al., 2015). This hypothesis relies on simple ecological assumptions. The vegetation quantity hypothesis proposes that higher stand biomass implies greater light absorption and water interception, which in turn foster ecosystem productivity (Lohbeck et al., 2015; Yue et al., 2022). This is particularly relevant when considering research showing short-term N addition increases productivity by promoting plant growth rather than biodiversity in nitrogen-constrained ecosystems (Bedison and McNeil, 2009; Han et al., 2011; Zaehle et al., 2011). Taken together, these results imply that resource effects may be bigger than biodiversity effects, further strengthening the need to research ecological interactions that affect resource availability (such as fire and N-deposition) (Wang et al., 2022).

The Yellow River Delta (YRD) is an extremely saline coastal wetland located in North China. Halophytes are widely distributed in this area, and *Tamarix chinensis* is the dominant species of shrub layer. The habitats in the YRD are lean, heterogeneous, and fragile and are extremely sensitive to external disturbances, such as fire and N deposition. According to previous records, the N deposition rate in the YRD has reached 2.3 g N m^−2^ year^−1^, a relatively high level in China (Yu et al., 2014). Studies suggest the deposition of a high N concentration can increase the nutrients in the soil and enhance plant production (Guan et al., 2019) but reduce the diversity of soil microorganisms (Lu et al., 2021) in the YRD coastal wetland ecosystem.

To examine the interaction of fire and N addition (simulated N deposition), a field experiment in *T. chinensis* communities was conducted in the YRD. Fire and N addition treatments were carried out, and 1 year later, soil physicochemical properties, soil microbial biodiversity, aboveground herbaceous biodiversity, and aboveground productivity were measured. We hypothesized the following: First, N addition would suppress the stimulating effect of fire on both herbaceous biodiversity and soil microbial biodiversity. Secondly, N addition would increase herbaceous aboveground productivity. Thirdly, productivity would be governed by different factors in plots treated with fire *vs*. those that had not burned.

## 2 Materials and methods

### 2.1 Site description

**Figure 1:**
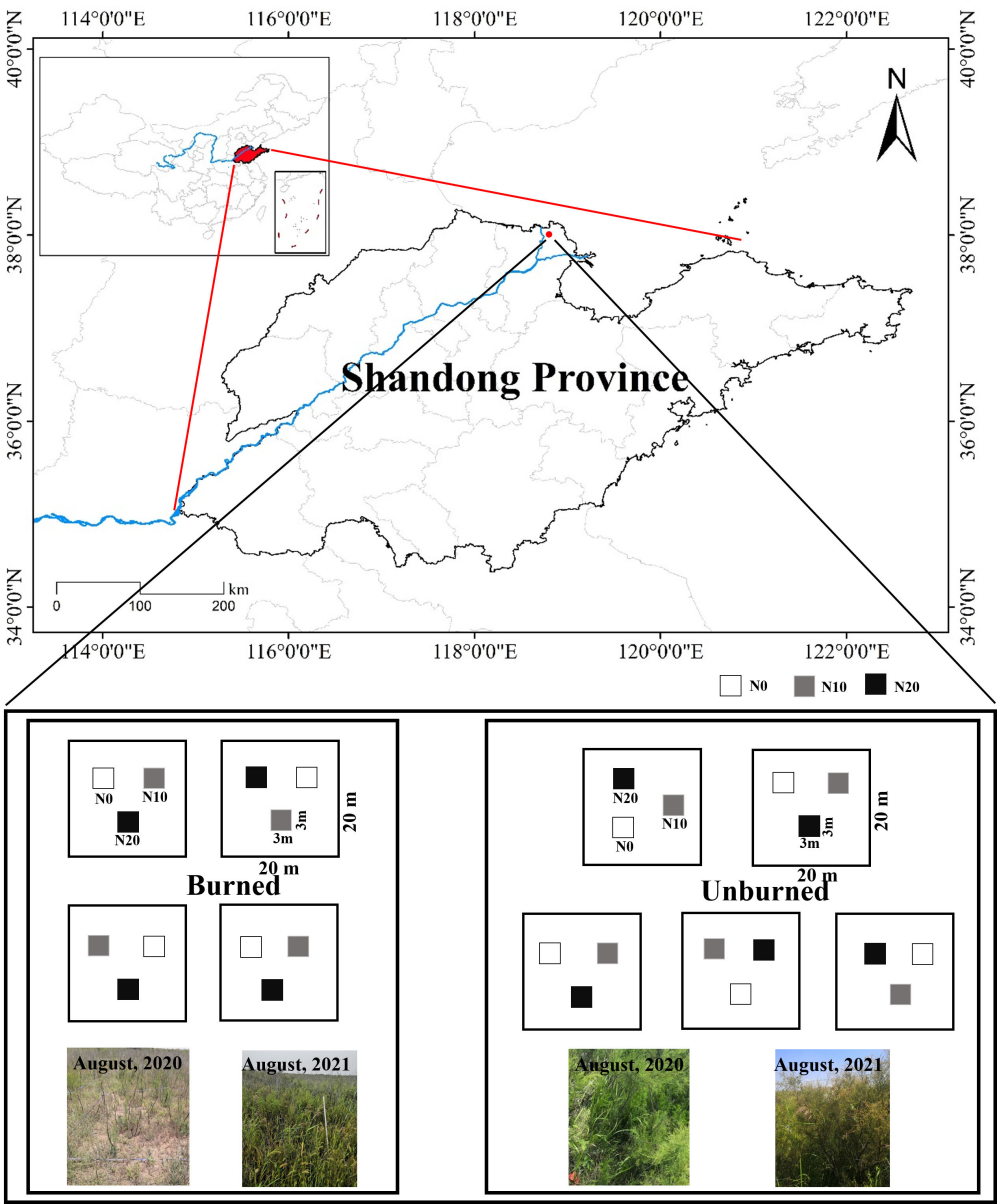
Location of the studied area and the experimental design.

The study area is located in the Yellow River Delta in Dongying, Shandong Province, China (38° 2′ 29″ N, 118° 44′ 42″ E) (**Figure 1**). This region has a typical warm-temperate and semi-humid continental monsoon climate. It has distinct seasons with an annual average temperature of 12.3°C and annual average precipitation of 542 mm, approximately 75% of which occurs from May to September (Liang et al., 2021), so it is typified by distinct dry and wet seasons.

### 2.2 Experimental design

A typical and uniform shrubland dominated by *T. chinensis* and recently burned was selected for analysis in August 2020. Judging by depth, the fire was a low-severity fire, and there was approximately 2.5 cm ± 0.4 cm of burned organic soil (Kasischke et al., 2008). We identified two plots (one burned, and one unburned) within 30 m of each other for study. In this way, the plant community composition and soil characters were kept as identical as possible for the plots prior to the fire incident. We observed that old branches of *T. chinensis* were carbonized in the fire, and new branches were sprouting from the base of mother individuals. The dominant herbaceous species in both plots was *Aeluropus sinensis*. An N addition treatment was applied in both the unburned and burned areas. Three N addition levels (N0: 0 g N m^−2^ year^−1^, N10: 10 g N m^−2^ year^−1^, N20: 20 g N m^−2^ year^−1^) were used in our study to represent both current rates of N deposition rate and predicted deposition rates representing the near future. N was added as NH_4_NO_3_ quarterly in August 2020. In May and August, NH_4_NO_3_ solution was prepared with pure water and sprayed evenly into different plots. In November and February, NH_4_NO_3_ was added in powder form but mixed with sand in advance. The N0 treatments were added the same amount of pure water or sand without NH_4_NO_3_. Five blocks of 20 m × 20 m were chosen in the unburned area, and four blocks of 20 m × 20 m were chosen in the burned area on account of the size limitation of the burned area. In each block, three plots (3 m × 3 m) were fixed and randomly designed as the three N addition levels. The blocks were at least 10 m apart from each other, and the plots were separated by a more than 2-m buffer zone (**Figure 1**). All the blocks and plots were well managed and protected from any damage and disturbance during the experiment.

### 2.3 Field investigation

Field investigation and sample collection proceeded on 27 August 2021, after about 1 year of fire recovery and N addition. For the shrubs, we recorded the individual number, total coverage, maximal growth height, and average growth height. Maximal height (Shrub *H*_max_) was measured as the average distance between the highest photosynthetic tissues and the ground (Yi et al., 2021). The shrub’s average height was the height of *T. chinensis* and represents the mean height in the plot. For the herb layer, we recorded the taxonomic identity, the number of every species, and aboveground net primary productivity (ANPP). The average height of the herbaceous layer (*H*_average_) and several biodiversity indices (Patrick richness index, Shannon–Wiener diversity index, and Pielou’s evenness index) were then calculated as follows: ANPP was collected using a 0.5 m × 0.5 m quadrate, which was randomly placed in each plot as well as 30 cm inside the border of each plot to avoid edge effects.

Herbaceous H_average_:


$${\text{H}}_{\text{average}}=\frac{{{\text{H}}^{\prime }}_{\text{average}}{\times \text{Number}}_{\text{i}}}{\text{Total number}}$$

Patrick richness index:


R=S

Shannon–Wiener diversity index:


$${\text{H}}^{\prime }=-\sum_{\text{i}=1}^{S}{\text{P}}_{\text{i}}\ln\left({\text{P}}_{\text{i}}\right)$$

Pielou’s evenness index:


$$\text{J}=\frac{H\prime }{\text{ln}\left(S\right)}$$

Where *H*_average_ is the average height of the herbaceous layer, *H*′_average_ is the average height of each species, Number*_i_* is the number of each species, Total number is the total number of species in the plot, *S* is the total number of species in the plot, and *P_i_* is the proportion of the number of individuals of species *i* to the total number of individuals in the plot (Ricotta and Avena, 2003).

### 2.4 Soil sampling and physicochemical property analysis

Soil water content (*W*) and temperature (*T*) of surface soils (0 cm–10 cm) were measured using a soil sensor reader (Item-6466, Spectrum, Aurora, America) in the field. Soil samples (0 cm–20 cm depth, 4 cm diameter) were collected following a five-point sampling method at the same time as the plant communities’ investigation. After being cold-preserved for transport, each soil sample was divided into two parts. One part was stored in a refrigerator at −80°C for microbial sequencing, and the other part was air-dried and sieved through a 2-mm mesh to determine several abiotic soil properties, including soil pH (pH), soil electrical conductivity (EC), soil total nitrogen content (TN), soil total phosphorus content (TP), and soil organic carbon content (SOC). pH was measured using a pH meter (FE28-Standard, Mettler Toledo, Shanghai, China). EC was measured using a conductivity meter (FE38-Standard, Mettler Toledo, Shanghai, China). TN was measured by the Kjeldahl digestion method using an automatic azotometer (K9860, Hanon, Jinan, China). TP was measured using the Mo–Sb colorimetric method using a spectrophotometer (UV-9100, Unico, Hunan, China). SOC was measured by dichromate titration. The carbon–nitrogen ratio (SOC/TN) and nitrogen–phosphorus ratio (TN/TP) were calculated subsequently.

### 2.5 16S rRNA gene and ITS amplicon sequencing

16S rRNA gene and ITS high-throughput sequencing were performed by OE Biotech Co. Ltd. (Shanghai, China), using an Illumina MiSeq platform. Nonconserved regions (16S V3V4) of 16S rRNA genes were amplified using the 343F primer (TACGGRAGGCAGCAG) and the 798R primer (AGGGTATCTAATCCT). Fungal ITS analysis was done using ITS1 (5′-CTTGGTCATTTAGAGGAAGTAA-3′) and ITS2 (5′-GCTGCGTTCTTCATCGATGC-3′) primers. To ensure the quality of downstream analysis, the raw data from Illumina MiSeq sequencing was processed to remove ambiguous bases of paired-end reads. Low-quality sequences with an average quality score of less than 20 and sequences of< 50 bp were removed. Subsequently, the paired-end reads were spliced at a minimum overlap of 10 bp, a maximum overlap of 200 bp, and a maximum mismatch rate of 20%. QIIME (QIIME 2 Development Team, USA) and UCHIME (UCHIME 2.4.2, Tiburon, CA, USA) were used to remove N bases and reads shorter than 200 bp and reads with chimeras, respectively. Clean reads were clustered to generate OTUs using Vsearch (Vsearch 2.4.2, San Diego, CA, USA) using a 97% similarity cutoff. The most abundant sequence in each OTU was selected as the representative sequence of the OTU. Subsequently, BLAST was used to annotate the representative sequence. At the same time, we also got alpha diversity (Chao 1, Shannon–Wiener index, and Simpson index). Unidentified sequences and nonmicrobial sequences were classified as “unidentified” for further analysis. Finally, phyla and genera with > 1% abundance were graphed, while phyla and genera whose abundance was less than 1% were grouped as “Others”.


$$\text{Chao }1=S+\frac{{\text{n}}_{1}({\text{n}}_{1}-1)}{2\left({\text{n}}_{2}-1\right)}$$

$$\text{Shannon–Wiener index}=-\sum_{i-1}^{s}\frac{{\text{n}}_{\text{i}}}{\text{N}}\ln\frac{{\text{n}}_{\text{i}}}{\text{N}}$$

$$\text{Simpson index}=1-\sum_{i=1}^{S}\frac{{\text{n}}_{\text{i}}\left({\text{n}}_{\text{i}}-1\right)}{\text{N}\left(\text{N}-1\right)}$$

Where *S* is the number of species observed, *n*_1_ is the number of OTUs with only one sequence, *n*_2_ is the number of OTUs with two sequences, *N* is the total sequences of the bacterial or fungal sample, and *n_i_* is the number of sequences in group *i*.

### 2.6 Statistical analysis

A two-way analysis of variance (ANOVA) was performed using IBM’s SPSS Statistics (SPSS Inc., Chicago, USA) software to detect the main effects and the interaction of fire and N addition. Duncan’s test was used for multiple comparisons, and the test level *α* = 0.05 was set. All data were tested for normality and homogeneity of variance before ANOVA, and log or square root transformations were performed when necessary. Box plots, grouping bar charts, and histograms at the phyla level of the microbial community were drawn using OriginPro 2021 (Originlab Co., Northampton, MA, USA) software. The “ggplot2” package in R Statistical Software v. 4.1.3 (R Core Development Team, 2020) was used for principal component analysis (PCA).

## 3 Results

### 3.1 Effects of fire and nitrogen addition on soil properties

**Figure 2:**
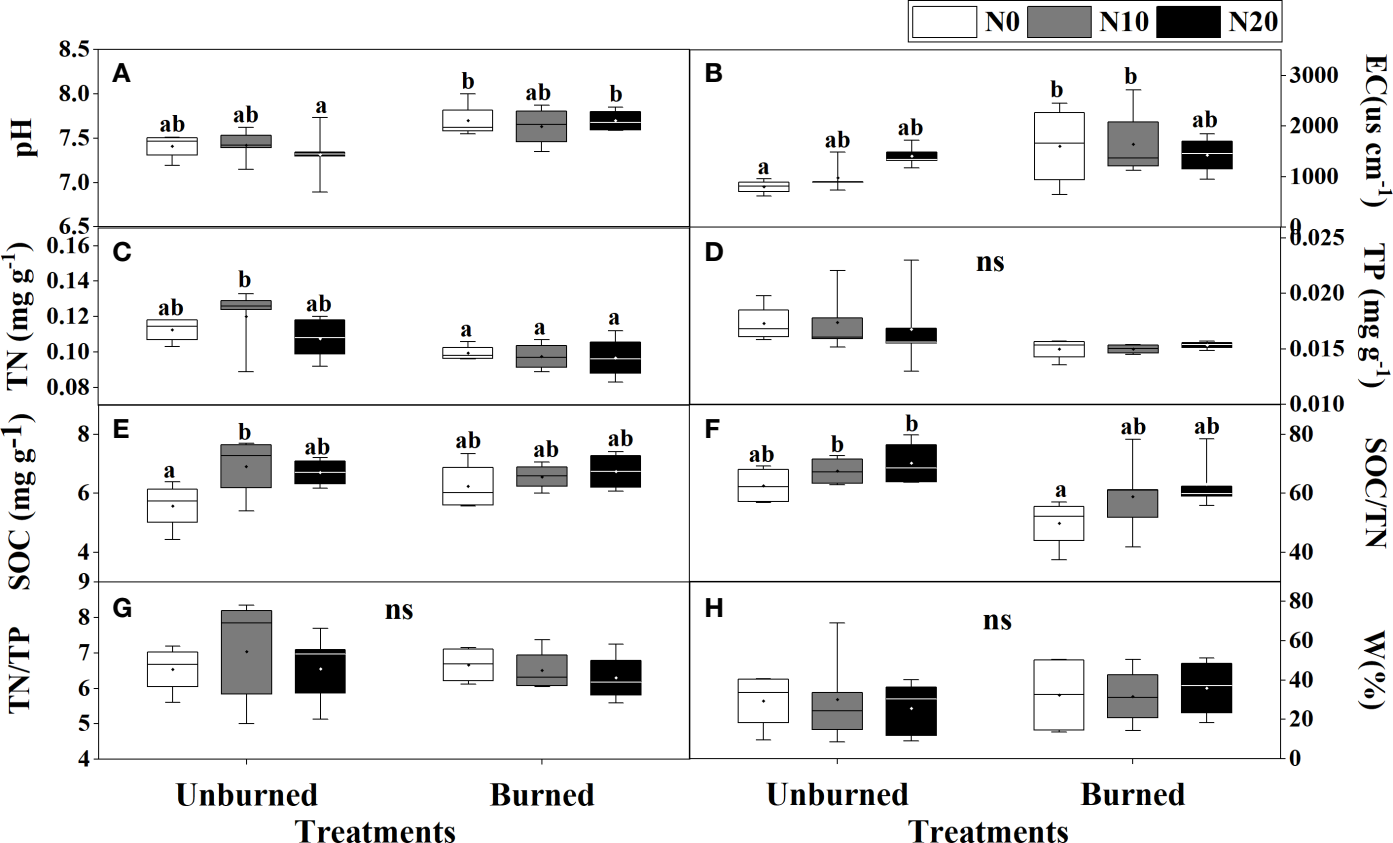
Soil physicochemical properties **(A–H)** after fire (unburned vs. burned) and N addition (N0: 0 g N m^−2^ year^−1^, N10: 10 g N m^−2^ year^−1^, N20: 20 g N m^−2^ year^−1^) treatments for 1 year. Different letters indicate significant differences among different treatments, according to Duncan’s test (*p<* 0.05). ns indicates no significant differences among different treatments. The values of the boxplot were the original data of four to five replicates. The abbreviations are the same as **Table 1**.

Fire had a significant effect on soil pH, EC, TN, TP, and SOC/TN (*p<* 0.05) (**Table 1**). Soil pH and EC were higher in burned plots than in unburned plots (**Figures 2A, B**). Both soil TN and TP were reduced by burning, and burning had obvious effects on TN (**Table 1**, **Figures 2C, D**). SOC, TN/TP, and W were the same in burned and unburned plots (**Table 1**, **Figures 2E, G, H**).

**Table 1:** The *F*-values under fire (unburned vs. burned) and N addition (0 g N m^−2^ year^−1^, 10 g N m^−2^ year^−1^, 20 g N m^−2^ year^−1^) treatments on soil properties.

Index	Burning	N addition	Burning × N addition
pH	12.764^**^	0.101	0.374
EC (μs cm^−1^)	6.858^*^	0.428	1.649
TN (mg g^−1^)	11.461^**^	0.768	0.672
TP (mg g^−1^)	5.464^*^	0.008	0.126
SOC (mg g^−1^)	0.162	3.260	0.953
SOC/TN	7.167^**^	2.877	0.224
TN/TP	0.335	0.303	0.252
W (%)	0.505	<0.001	0.155
T (°C)	0.155	0.150	0.150

The numbers in the table were F-values, repetitions n = 4–5. The significance of each effect was expressed in bold font “*”. ^*^p< 0.05; ^**^p< 0.01; ^***^p< 0.001. pH, soil pH; EC, soil electrical conductivity; TN, soil total nitrogen content; TP, soil total phosphorus content; SOC, soil organic carbon content; W, soil water content; T, soil temperature.

Nitrogen addition did not influence most soil characteristics regardless of burning treatments (**Table 1**, **Figure 2**). Interestingly, SOC increased with N addition in unburned plots, and SOC/TN increased in burned plots (**Figures 2E, F**). The interaction between fire and N addition had no significant impact on soil physical and chemical properties (**Table 1**).

### 3.2 Effects of fire and nitrogen addition on plant traits and plant community parameters

**Figure 3:**
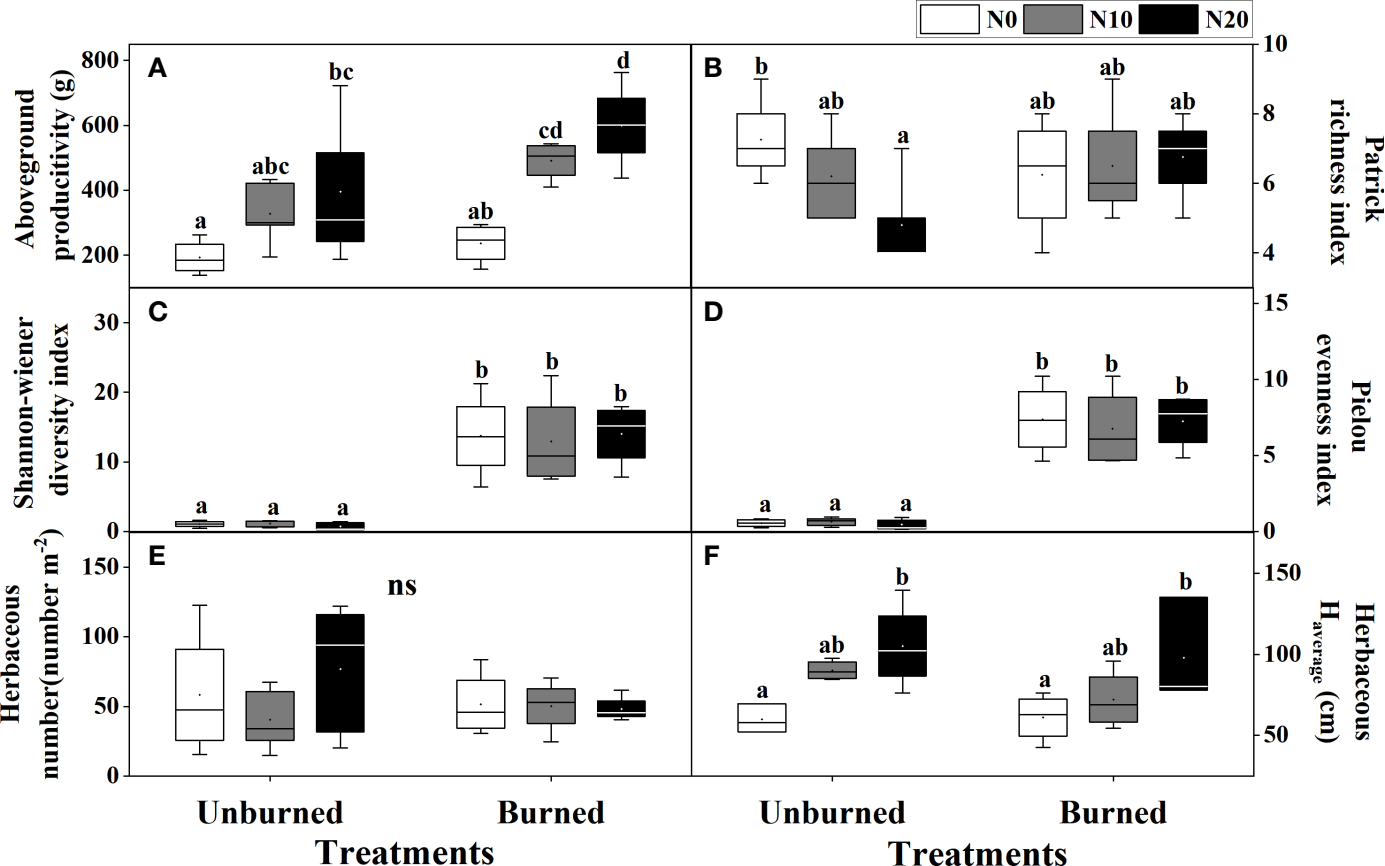
Herbaceous traits **(A–F)** after fire (unburned vs. burned) and N addition (N0: 0 g N m^−2^ year^−1^, N10: 10 g N m^−2^ year^−1^, N20: 20 g N m^−2^ year^−1^) treatments for 1 year. Different letters indicate significant differences among different treatments, according to Duncan’s test (*p<* 0.05). ns indicates no significant differences among different treatments. The values of the boxplot were the original data of four to five replicates. The abbreviations are the same as **Table 1**.

Both fire and N additions had significant impacts on the aboveground productivity of the herbaceous layer (*p<* 0.05) (**Table 2**). Compared with N0 treatment, aboveground productivity in N10 and N20 increased 107.76% and 153.81% in burned plots and 70.35% and 105.38% in unburned control plots, respectively (**Figure 3A**). At each nitrogen addition level, aboveground productivity under burned conditions was higher than that under unburned conditions.

**Table 2:** The *F*-values under fire (unburned vs. burned) and N addition (0 g N m^−2^ year^−1^, 10 g N m^−2^ year^−1^, 20 g N m^−2^ year^−1^) treatments on plant growth, biodiversity, and productivity.

Index	Burning	N addition	Burning × N addition
Herbaceous productivity and biodiversity			
Aboveground productivity (g)	7.565^*^	10.958^**^	0.905
Patrick richness index	0.546	0.998	2.265
Shannon–Wiener diversity index	64.032^***^	0.023	0.080
Pielou’s evenness index	113.994^***^	0.064	0.144
Herbaceous individual number	0.446	0.634	0.785
Herbaceous *H*_average_ (cm)	0.912	7.496^**^	0.446
*Tamarix chinensis* growth			
Shrub individual number (number plot^−1^)	4.861	1.267	0.815
Cover (%)	36.935^***^	1.571	0.109
Shrub *H*_max_ (cm)	38.361^***^	0.585	0.194
Shrub *H*_average_ (cm)	82.035^***^	2.063	0.355

The numbers in the table were F-values, repetitions n = 4–5. The significance of each effect was expressed in bold font “*”. ^*^p< 0.05; ^**^p< 0.01; ^***^p< 0.001. Herbaceous H_average_, herbal average height; Shrub H_max_, the shrub maximum height; Shrub H_average_, the shrub average height.

The average height of herb species significantly increased with N addition under both burned conditions (*p<* 0.05) (**Figure 3F**). Moreover, compared with the unburned condition, fire significantly increased the Shannon–Wiener diversity index and Pielou’s evenness index, but the effect of N addition was not significant (*p<* 0.05) (**Table 2**, **Figures 3C, D**). Interestingly, in unburned conditions, the Patrick richness index (number of species) of herbs significantly decreased with the increase in N addition (**Figure 3B**). There was no significant difference in herbaceous individual numbers among different treatments (**Table 2**, **Figure 3E**). Moreover, no significant impact on herbaceous aboveground productivity and biodiversity was found under the interaction between fire and N addition treatment (**Table 2**).

**Figure 4:**
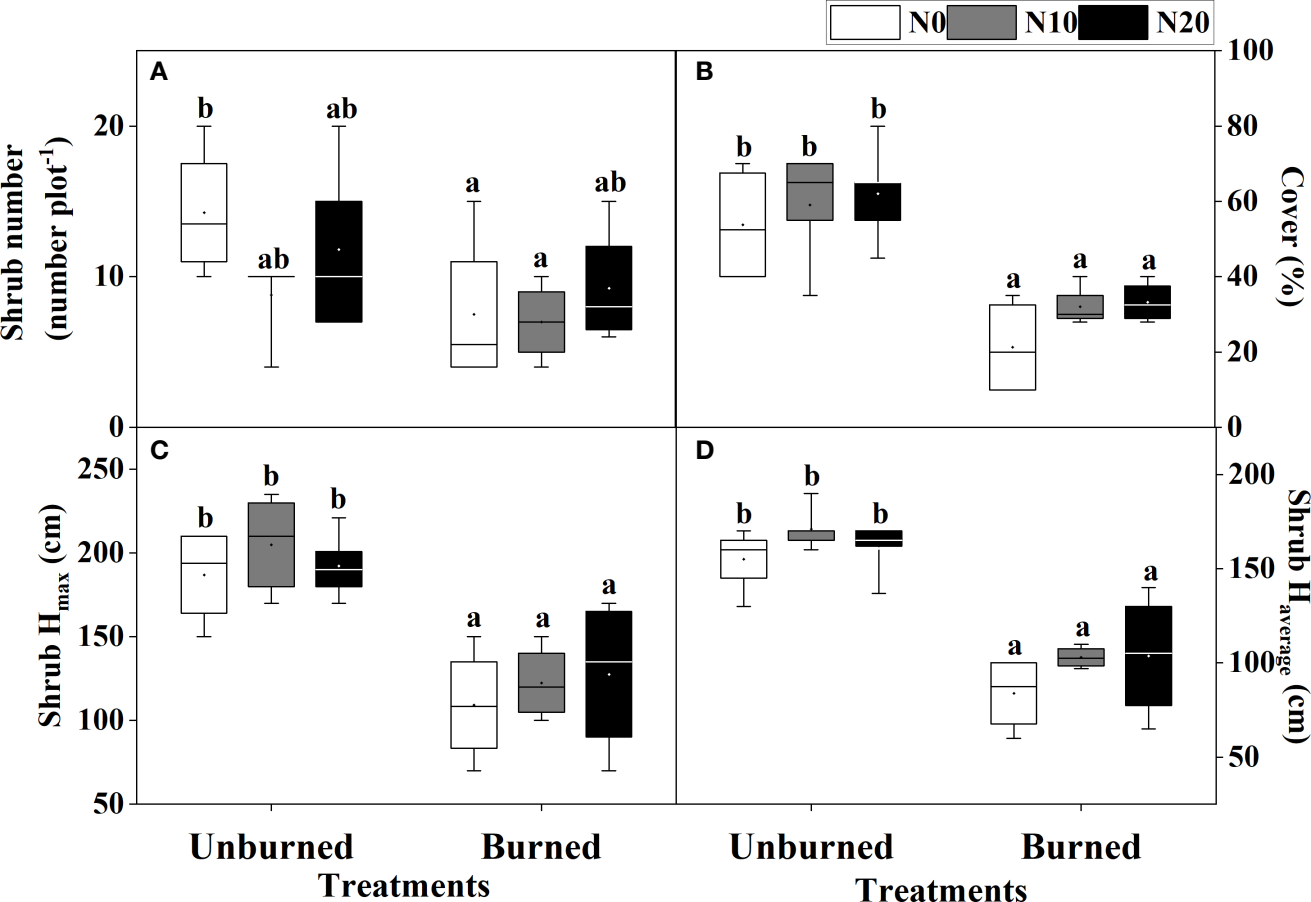
Growth of *T. chinensis* **(A–D)** after fire (unburned vs. burned) and N addition (N0: 0 g N m^−2^ year^−1^, N10: 10 g N m^−2^ year^−1^, N20: 20 g N m^−2^ year^−1^) treatments for 1 year. Different letters indicate significant differences among different treatments, according to Duncan’s test (*p<* 0.05). The values of the boxplot were the original data of four to five replicates. The letter meaning and unit are the same as in **Table 1**.

Fire significantly reduced coverage, *H*_max,_ and *H*_average_ of *T. chinensis* (*p<* 0.05) (**Table 2**, **Figures 4B–D**). However, the actual number of *T. chinensis* individuals was only impacted by fire under the N0 condition (**Figure 4A**). No significant impact of N addition on shrub growth could be identified in either fire treatment.

### 3.3 Effects of fire and nitrogen addition treatments on soil microbial biodiversity

**Figure 5:**
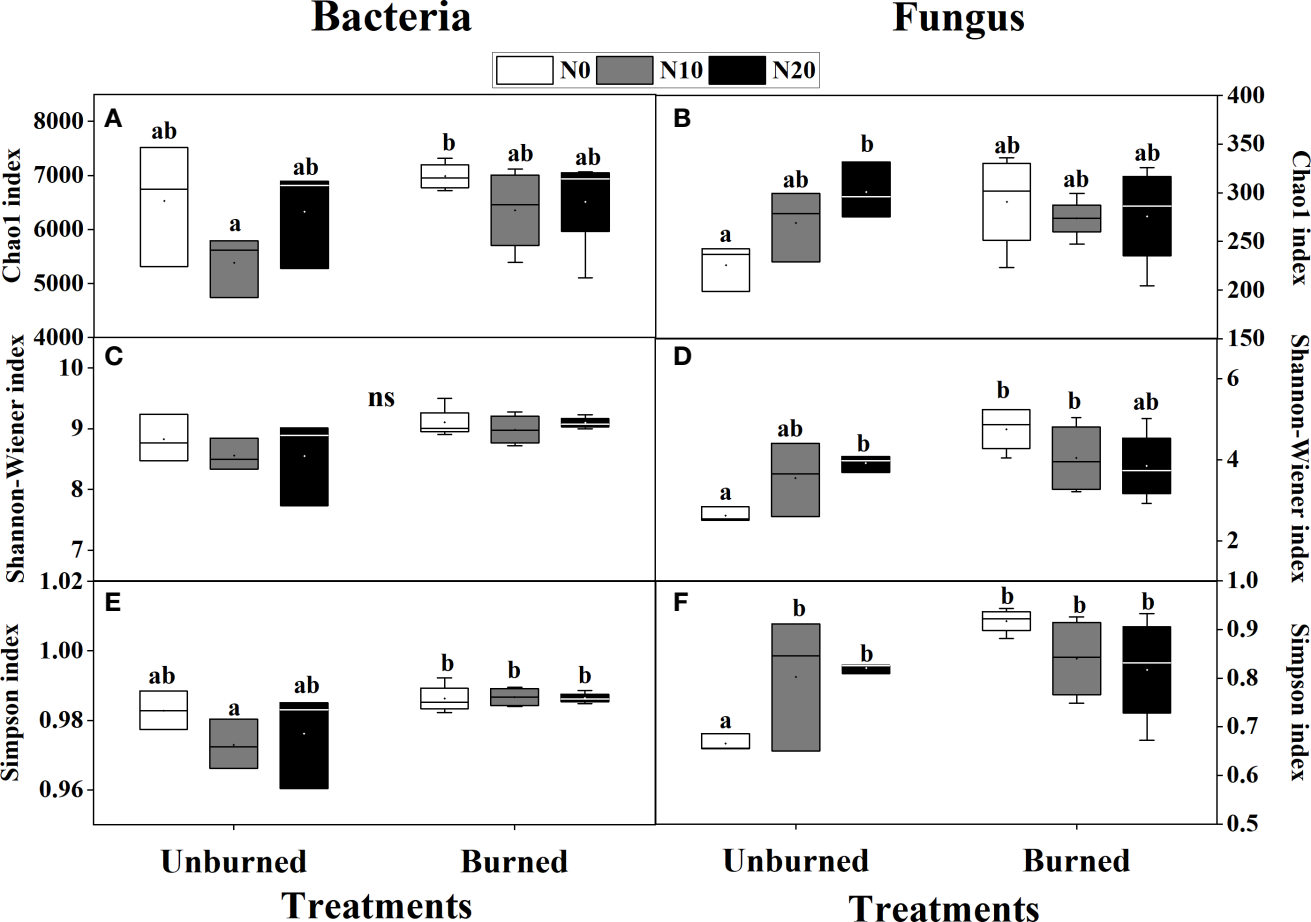
Bacterial and fungal biodiversity index after fire (unburned vs. burned) and N addition (N0: 0 g N m^−2^ year^−1^, N10: 10 g N m^−2^ year^−1^, N20: 20 g N m^−2^ year^−1^) treatments for 1 year. **(A)** Bacterial Chao1 index; **(B)** fungal Chao1 index; **(C)** bacterial Shannon–Wiener index; **(D)** fungal Shannon–Wiener index; **(E)** bacterial Simpson index; **(F)** fungal Simpson index. Different letters indicate significant differences among different treatments, according to Duncan’s test (*p<* 0.05). The values of the boxplot were the means of three to four replicates.

Fire had a significant effect on both the Shannon-Wiener and Simpson indices of both bacterial and fungal alpha diversity (*p<* 0.05) (**Table 3**). Bacterial alpha diversity did not vary among N addition treatments (**Figures 5A, C, E**). Interestingly, the alpha diversity of fungi increased with N addition in the unburned control plots, and this pattern was not observed in the burned plots (**Figures 5B, D, F**).

**Table 3:** The *F*-values under fire (unburned vs. burned) and N addition (0 g N m^−2^ year^−1^, 10 g N m^−2^ year^−1^, 20 g N m^−2^ year^−1^) treatments on microbial α diversity.

Group	Index	Burning	N addition	Burning × N addition
**Bacteria**	Chao 1 index	2.310	2.149	0.432
	Shannon–Wiener index	7.197^*^	0.554	0.245
	Simpson index	10.311^**^	0.963	1.130
**Fungus**	Chao 1 index	0.719	1.011	2.327
	Shannon–Wiener index	7.223^*^	0.124	4.320^*^
	Simpson index	6.886^*^	0.279	4.741^*^

The numbers in the table were F-values, repetitions n = 3–4. The significance of each effect was expressed in bold font “*”. ^*^p< 0.05; ^**^p< 0.01.

**Figure 6:**
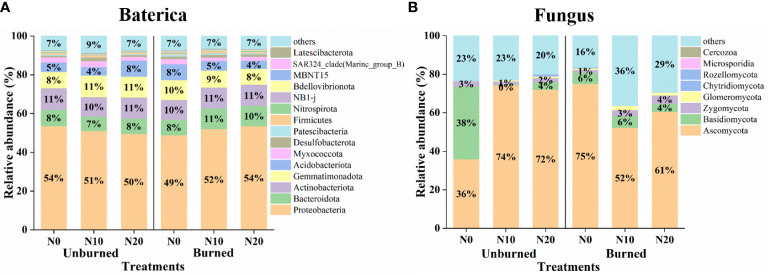
Microbial community structure at the phylum level after fire (unburned vs. burned) and N addition (N0: 0 g N m^−2^ year^−1^, N10: 10 g N m^−2^ year^−1^, N20: 20 g N m^−2^ year^−1^) treatments for 1 year. **(A)** Bacterial community structure of phylum level. **(B)** Fungus community structure of phylum level.

Generally, fire and N addition had no significant effect on the relative abundance of bacterial communities (**Figure 6A**). In terms of relative abundance, the top 10 bacterial phyla were Proteobacteria, Actinobacteriota, Gemmatimonadota, Bacteroidota, Acidobacteriota, Myxococcota, Desulfobacterota, Firmicutes, Patescibacteria, and Nitrospirota. Proteobacteria was the most dominant phylum regardless of either N addition or burning. We also obtained the top eight fungal phyla, including Ascomycota, Basidiomycota, Zygomycota, Glomeromycota, Rozellomycota, Chytridiomycota, Microsporidia, and Cercozoa. The dominant phylum was always Ascomycota under all treatments (**Figure 6B**).

### 3.4 Factors explaining herbaceous aboveground productivity

**Figure 7:**
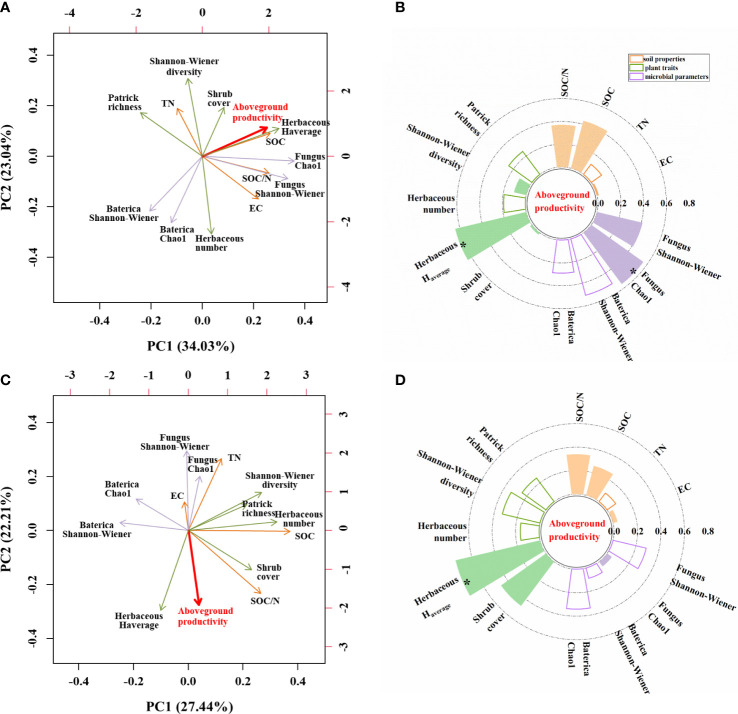
The influence of soil, plant, and microbial parameters on herbaceous aboveground productivity under unburned **(A, B)** and burned conditions **(C, D)**. **(A, C)** Principal component analysis (PCA) of herbaceous aboveground productivity and other indicators. The red line vectors represent herbaceous aboveground productivity; the green line vectors represent plant traits; and the purple line vectors represent microbe parameters, while the orange line vectors represent soil properties. **(B, D)** Grouping bar chart showing the correlation between herbaceous aboveground productivity and other indicators. Solid columns showed a positive correlation. Hollow columns showed a negative correlation. EC, soil electrical conductivity; TN, soil total nitrogen content; SOC, soil organic carbon content; Herbaceous *H*_average_, herbaceous average height; Number, herbaceous total number/plot. The significance of each effect is expressed in bold font; ^*^ *p<* 0.05.

We performed two PCA analyses to identify which environmental parameters could best explain variation in aboveground herbaceous productivity (**Figure 7**). The first two axes explained 57.07% of the variation under unburned conditions (**Figure 7A**) but only 49.65% of the variation under burned conditions (**Figure 7C**). In unburned plots, five parameters had correlation coefficients greater than 0.4, and only two parameters exceeded this threshold in burned plots. Herbaceous aboveground productivity was significantly positively correlated with herbaceous average height and fungal Chao 1 in unburned fields (**Figures 7A, B**). In burned fields, however, herbaceous aboveground productivity was only correlated with herbaceous average height (**Figures 7C, D**).

## 4 Discussion

### 4.1 N addition could suppress herbaceous richness but improve fungal biodiversity

N addition reduced herbaceous richness by improving the competitive ability of the dominant species. In unburned plots, the Patrick richness index decreased for herbaceous species with increasing levels of N addition (**Figure 3B**). In our study, we found that the height of the main dominant species of the community, such as *S. glauca* and *P. australis*, was significantly promoted by N addition (*p*< 0.05). This suggests that nutrient addition allowed the dominant species to compete better and grow better, which fits the theory of “The Matthew Effect”. As the dominant species grew better, it crowded out subordinate or transient species, which may explain why some species such as *Lactuca tatarica*, *Takhtajaniantha austriaca*, and *Sonchus brachyotus* were excluded under N addition. In this manner, height may also affect ecosystem productivity by measuring the impact of improved competition on biodiversity. We found that herb richness was negatively correlated with herb height under different fire conditions (**Figures 7A, C**), which might reflect the influence of competition effects of species for light resources. Plant competition strategy suggests the competition of plants for aboveground light is asymmetrical (Schwinning and Weiner, 1998). Fertilization increased the content of available resources in the soil, which reduced underground competition for resources but ultimately may have increased aboveground competition for light. Ren et al. (2010) considered that the competition between species for the lower layer of light was one of the main reasons for the decrease in species biodiversity caused by resource addition. Unfortunately, we found that N addition could not mitigate the effect of fire on biodiversity (herbaceous biodiversity and soil microbial biodiversity) in our study.

The carbon-to-nitrogen ratio may be the main factor affecting fungal diversity under unburned plots. We found that N addition significantly increased the biodiversity of the fungi community in unburned plots (**Figures 5B, D, F**). It is possible that N addition improved the C/N ratio, which is associated with fungal enrichment (Bahram et al., 2018). Our results more strongly showed that SOC, SOC/TN (**Figures 2E, F**), and fungal diversity index (**Figures 5B, D, F**) increased with N addition under unburned plots. Furthermore, fungal alpha diversity (both Chao 1 and Shannon–Wiener) was positively associated with the ratio of SOC to TN (**Figure 7A**). Taken together, these results suggest that the distribution of fungi is limited by resource availability, corresponding to their greater energy demand (Bahram et al., 2018). This finding also suggests the dominance of fungi over bacteria in N-limited soils benefits C sequestration. An increase in fungal dominance has been reported to increase organic carbon accumulation in the soil as the biomass and residues of fungi have higher resistance to decomposition. Furthermore, fungi may contribute to the formation of macroaggregates that retain carbon in entangled soil particles (Cui et al., 2022).

### 4.2 Fire increased herbaceous biodiversity and soil microbial diversity

We observed that fire increased the Shannon diversity index and Pielou’s evenness index of herbaceous species (**Figures 3C, D**). There are many reasons why fire may improve plant biodiversity. Firstly, fire provides understory species the opportunity to emerge by removing dominant upper-layer species (He et al., 2019). This is further supported when we consider that our dominant herbaceous species are R-strategy annual halophytes (except *Phragmites australis*), characterized by quick growth and a high level of tolerance to saline soils (Pierce et al., 2017). Secondly, low-severity fires also promote germination by increasing illumination and removing dry litter (Pereira et al., 2016). We observed that fire significantly decreased plant coverage and growth height in *T. chinensis*, the only shrub species in our study area (**Table 2**, **Figure 4**), and posit that the increased black ash cover may change soil thermal properties in the post-fire period (e.g., by reducing the albedo and capturing sun radiation). These changes in soil temperature dynamics can modify soil daily temperatures to the point of breaking physical seed dormancy and promoting seed germination (Santana et al., 2010). All in all, an appropriate fire regime may increase plant biodiversity in the coastal wetlands.

Theoretically, fire disturbance causes microbial death by heating the soil, and thus fire is associated with changing the composition of soil microbial communities (Pressler et al., 2019). However, in our study, fire increased the Shannon index and Simpson index of both bacteria and fungus (**Table 3**, **Figures 5C, D**), contrary to several previous studies (Nelson et al., 2022). We believe that recovery time after a fire should be taken into account as an essential explanatory variable. For example, fires are known to negatively impact biomass, activity, and diversity of microorganism communities in the short term (1–3 months after a fire) (Barreiro and Diaz-Ravina, 2021). However, fire has also been attributed to long-term increases in bacterial and fungal biomass and diversity 2–3 years after a wildfire (Rodriguez et al., 2018). At even greater scales, fire has been attributed to the decrease in bacterial and fungal biomass after 14 years (Allam et al., 2020). This confirms that the resilient soil microbial population and diversity were able to adapt to the new environmental conditions caused by the wildfire. In addition, when considering the drivers of soil microbial communities in burned areas, it was necessary to combine vegetation community with postfire recovery time because vegetation dynamics and postfire recovery time were inseparable. That was to say, fire disturbance may change the plant-mediated changes of the soil environment by changing the composition of vegetation to realize indirect effects on soil microorganisms. Our results showed fire increased the Shannon–Wiener diversity index and Pielou’s evenness index of herbs (**Figures 3C, D**), which may have contributed to the observed increase in microbial biodiversity. Numerous studies have found that plant traits (de Vries et al., 2012), plant root exudates (Hu et al., 2018a), and plant community diversity (Chen et al., 2019) can explain microbial community composition and biomass. Heterotrophic microbes mainly rely on aboveground litter inputs and belowground root exudates to survive and grow (Huffman and Madritch, 2018). It is unsurprising that changes to the aboveground community would be reflected in the belowground community and vice versa.

### 4.3 Fire reduced the soil fungal associates on herbaceous aboveground productivity

Most terrestrial ecosystems are limited by nitrogen (Bai et al., 2010; Song et al., 2011). This is also true in the Yellow River Delta. Because N deposition increased the nutrient elements available to plants by adding nitrogen content in the soil, we observed that it improved the productivity of plant communities to a certain extent in the short term, consistent with several other studies (Luyssaert et al., 2008; Zaehle et al., 2011; Li et al., 2020). We also found that N addition improved the average height and aboveground productivity of herbaceous species (**Figures 3A, F**), consistent with several other previous studies (Friedrich et al., 2012; Grigulis et al., 2013; Kubert et al., 2019). Given these results, we would like to emphasize that appropriate N addition may be an effective tool to facilitate the revegetation of these coastal ecosystems, regardless of fire regime.

However, we observed that N addition could not mitigate the powerful influence of fire on regulating ecosystem productivity. We believed that N addition may reduce the N-loss experienced after a fire, and prevent the cascade of downstream interactions between plants, soil, and nitrogen that ultimately reduce productivity after a fire. This was untrue. However, we did observe an interaction between fire and belowground microbial communities that may highlight why fire reduces ecosystem productivity. In unburned plots, herbaceous plant productivity was positively associated with both plant height and belowground fungal alpha diversity (Chao 1) (**Figure 7B**). In burned plots, there was no relationship between soil microbial diversity and productivity (**Figure 7D**). This may suggest that fire damages the important relationships between plants and microorganisms and that the microbial community may need a longer time (more than 1 year) to recover and re-establish the relationship with the plant community. This may also explain the diminished influence of our N addition in the burned plots, as microbial symbionts play critical roles in the nitrogen acquisition of plants (Zak et al., 1994). Long-term ecosystem monitoring approaches must be considered in the future to better understand the pivotal influencing factors of productivity.

## 5 Conclusions

Our study showed that the effects of fire on soil properties and biodiversity were more significant than those of nitrogen addition in the coastal wetland dominated by *T. chinensis*. The combined effect of fire and N addition was marginally significant for fungus biodiversity, suggesting N addition for 1 year has limited capacity to mitigate the impact of fire. The main reason for affecting productivity is likely to be that the increase in resources caused by nitrogen addition directly promotes plant growth. In addition, we found that fire may reduce the influence of belowground fungal associates on herbaceous aboveground productivity, perhaps representing a fire-induced disturbance of the soil microbiome that will take longer than 1 year to recover.

## Data availability statement

The datasets presented in this study can be found in online repositories after January 1, 2024. The names of the repository/repositories and accession number(s) can be found below: BioProject, PRJNA996527.

## Author contributions

LQ: Conceptualization, Formal analysis, Data curation, Investigation, Writing - original draft, Visualization. YS: Data curation, Formal analysis, Writing - review & editing. PZ: Writing - review & editing, Data curation. WS: Writing - review & editing. WW: Funding acquisition, Data curation. SY: Funding acquisition, Investigation, Data curation. JL: Resources, Data curation, Investigation. HL: Resources, Data curation. ZB: Resources, Investigation. ND: Conceptualization, Funding acquisition, Writing - review & editing, Project administration. WG: Funding acquisition. All authors contributed to the article and approved the submitted version.
