# Supplementary figures and images for: The combined effect of fire and nitrogen addition on biodiversity and herbaceous aboveground productivity in a coastal shrubland
Source: Front Plant Sci. 2023 Aug 29;14:1240591. doi: 10.3389/fpls.2023.1240591 (PMC10497117; doi:10.3389/fpls.2023.1240591)

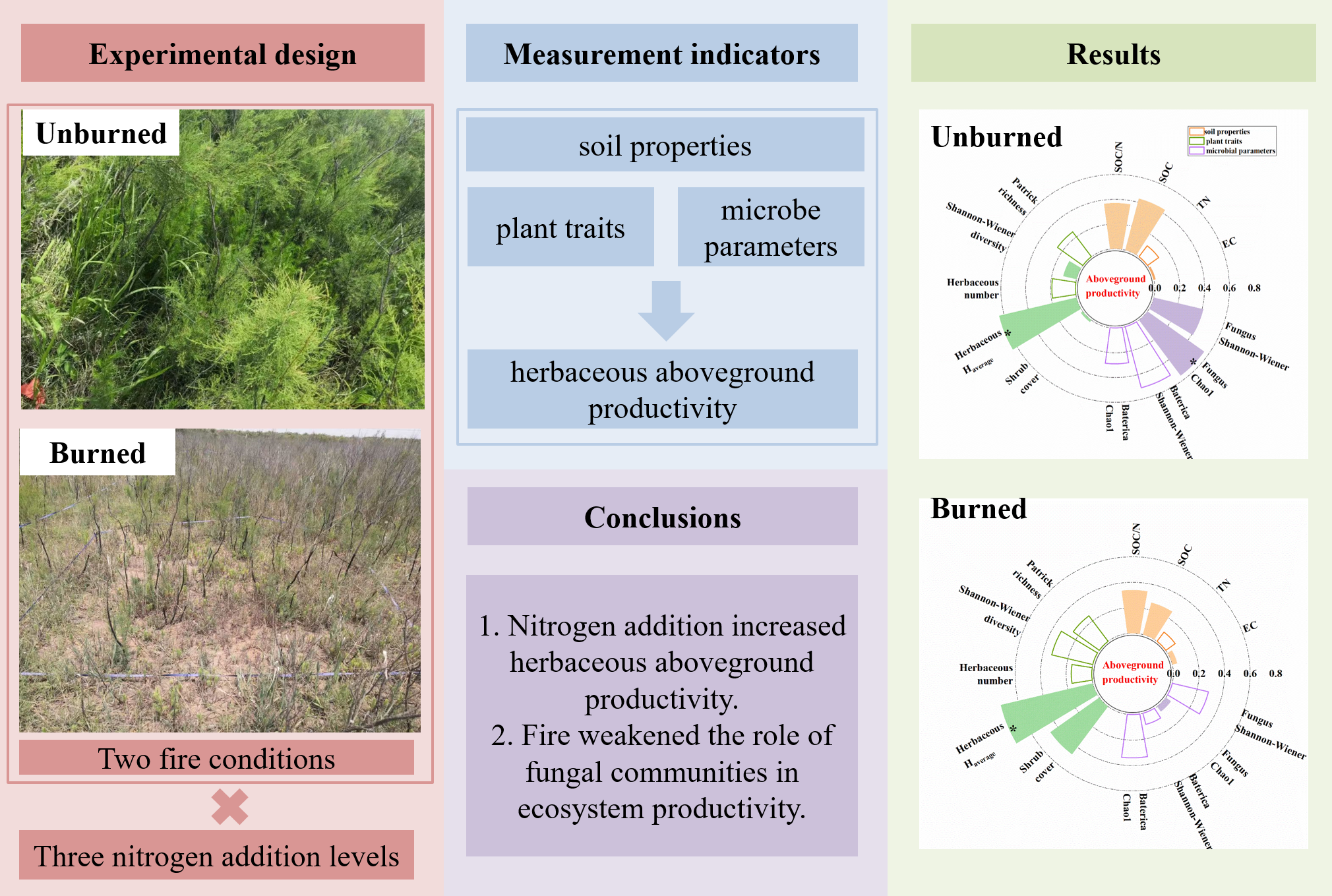

Supplement: Supplementary file 1 [file Image_1.tif]
